# Rosette-forming glioneuronal tumor: an illustrative case and a systematic review

**DOI:** 10.1093/noajnl/vdaa116

**Published:** 2020-09-09

**Authors:** Caleb P Wilson, Arpan R Chakraborty, Panayiotis E Pelargos, Helen H Shi, Camille K Milton, Sarah Sung, Tressie McCoy, Jo Elle Peterson, Chad A Glenn

**Affiliations:** 1 Department of Neurosurgery, University of Oklahoma Health Sciences Center, Oklahoma City, Oklahoma, USA; 2 Department of Pathology, University of Oklahoma Health Sciences Center, Oklahoma City, Oklahoma, USA

**Keywords:** case report, Rosette-forming glioneuronal tumor, systematic review

## Abstract

**Background:**

Rosette-forming glioneuronal tumors (RGNTs) are rare, low-grade, primary CNS tumors first described in 2002 by Komori et al. RGNTs were initially characterized as a World Health Organization (WHO) grade I tumors typically localized to the fourth ventricle. Although commonly associated with an indolent course, RGNTs have the potential for aggressive behavior.

**Methods:**

A comprehensive search of PubMed and Web of Science was performed through November 2019 using the search term “rosette-forming glioneuronal tumor.” Preferred Reporting Items for Systematic Reviews and Meta-Analyses (PRISMA) guidelines were followed. English, full-text case reports and series with histopathological confirmation were included. Patient demographics, presentations, MRI features, tumor location, treatment, and follow-up of all 130 cases were extracted.

**Results:**

A 19-year-old man with a history of epilepsy and autism presented with acute hydrocephalus. MRI scans from 2013 to 2016 demonstrated unchanged abnormal areas of cortex in the left temporal lobe with extension into the deep gray-white matter. On presentation to our clinic in 2019, the lesion demonstrated significant progression. The patient’s tumor was identified as RGNT, WHO grade I. One hundred thirty patients were identified across 80 studies.

**Conclusion:**

RGNT has potential to transform from an indolent tumor to a tumor with more aggressive behavior. The results of our systematic review provide insight into the natural history and treatment outcomes of these rare tumors.

**Key Points**

- Rosette-forming glioneuronal tumors have aggressive potential.
- While usually stable after resection, RGNT has been shown to occasionally recur.

**Importance of the Study**

Because the current literature on RGNT is still so limited, little is definitively understood about the epidemiology, anatomical distribution, and even treatment of these tumors, especially since there is a subset of cases with tumors that recurred. Not only does our case and review of the literature unveil the more aggressive clinical course that is possible with these tumors, but it also highlights other unique characteristics. For instance, our case involves fairly novel locations; there are only 13 other documented cases of the tumor in the third ventricle and only 2 others involving the temporal lobe. We also found that the gender preference, previously documented as at least a 2:1 females:males, was actually closer to 1:1 (68 females and 62 males). Other important findings are detailed in the manuscript.

The rosette-forming glioneuronal tumor (RGNT) is a rare, low-grade, primary CNS tumor that was first described in 2002 in a case series by Komori et al., in which the authors differentiated RGNT as a distinct entity from the dysembryoplastic neuroepithelial tumor.^1^ In 2007, the RGNT was designated as a World Health Organization (WHO) Grade I tumor and described as a slow-growing tumor typically localized to the fourth ventricle and composed of neurocytes that form neurocytic rosettes and glial components similar to those of the pilocytic astrocytoma.^2^ In the 2016 WHO classification, the RGNT was again classified as a Grade I tumor and listed under the category of “neuronal and mixed neuronal-glial tumors.” ^3^ Various other anatomical sites of origin have been reported, including the supratentorial ventricles,^4^ pineal gland,^5^ hypothalamus,^6^ optic chiasm,^7,8^ and spinal cord.^9–11^ Due to the rarity of the RGNT, little is known about its epidemiology and natural history. In this study, we present the case of a man with autism spectrum disorder and epilepsy who was diagnosed at 19 years of age with a RGNT. His prior MRI demonstrated stable appearance of nonspecific cortical abnormalities of the left temporal lobe that were thought to be related to his epilepsy. His case highlights the natural history of RGNT and presents a tumor in an uncommon anatomical location spanning from the temporal lobe into the basal ganglia extending to involve the cerebellum. In addition, we report the results of a systematic review of the literature pertaining to this rare neoplasm, analyzing key features of RGNT.

## Case Presentation

### Consent

Patient consent was obtained for inclusion in this case report. No identifying details have been disclosed.

### History and Physical Examination

A 19-year-old man with a history of autism spectrum disorder and simple partial epilepsy manifested by occasional generalized tonic-clonic seizures since childhood presented to our emergency department. He was first diagnosed with infantile spasms around the age of 3 months with up to 8 episodes per day and began antiepileptic therapy at that time. His childhood seizure semiology consisted of episodes of gaze deviation toward his right hand and right arm jerks, lasting 2–3 min. He also had repeated episodes of looking at his right hand, collapsing toward his right side, and heavy breathing. These episodes occurred 2–3 times weekly but were interspersed with asymptomatic periods lasting for several weeks. At the age of 6 years in 2005, the patient underwent an MRI during epilepsy workup, which revealed no pathological findings. Another MRI in 2013 demonstrated left temporal lobe cortical abnormalities with extension into the deep gray-white matter. The initial impression was focal hemimegaloencephaly of the mesial left temporal lobe with some polymicrogyria/pachygyria complex or possibly gliomatosis cerebri as read by neuroradiologists. There was no concern for neoplasm at the time. An MRI in 2016 showed the stable cortical lesions with a slight increase in the T2 signal in the superior cerebellar vermis.

**Figure 1.**
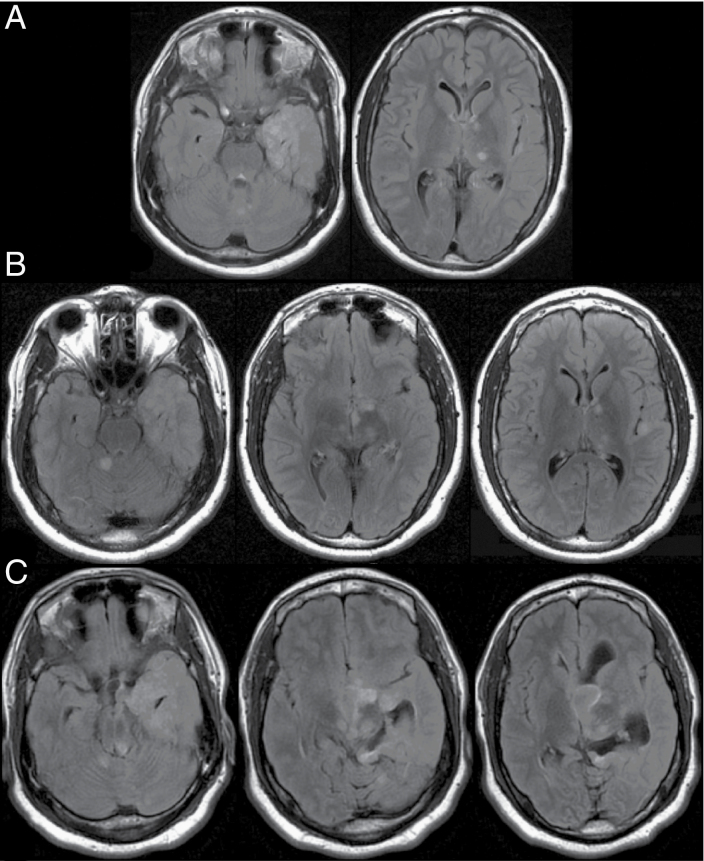
Axial Flair MRI demonstrating RGNT over a 6-year surveillance period. (A) 2013 MRI showing left temporal lobe cortical abnormalities with extension into the deep gray-white matter. (B) Surveillance MRI taken in 2016 that demonstrated stable cortical lesions with slight increase in the T2 signal in the superior cerebellar vermis. (C) MRI in 2019 at presentation to our institution with dramatic expansion of the previously stable left temporal lobe lesion with involvement of the left gangliocapsular region, bilateral thalami, tectum, and cerebellum. In addition, a new non-enhancing cystic component within the left foramen of Monro and third ventricle was identified, resulting in acute obstructive hydrocephalus.

In 2019, at age 19, the patient presented acutely with hydrocephalus manifested by a 2-week history of vomiting and progressive lethargy. Prior to this, he spent his days playing video games and was able to ambulate without difficulties. Upon presentation to the emergency room, he was very lethargic and had difficulties walking, repeatedly leaning, and falling toward his left side. MRI findings at this time showed dramatic expansion of the previously stable left temporal lobe lesion with involvement of the left gangliocapsular region, bilateral thalami, tectum, and cerebellum. In addition, a new nonenhancing cystic component within the left foramen of Monro and third ventricle was identified, resulting in acute obstructive hydrocephalus. The MRI scans from 2013, 2016, and 2019 are shown in Figure 1.

### Operative Course

On the day of presentation, an external ventricular drain (EVD) was placed to alleviate his hydrocephalus, resulting in mental status improvement. Shortly thereafter, an endoscopic biopsy of the third ventricle lesion along with septum pellucidotomy was performed. The initial evaluation of the pathology demonstrated concern for possible RGNT. However, only the rosette-forming neuronal component of the tumor was present within the nonenhancing third ventricular component. Given these results and the extensive nature of the tumor, a second procedure was planned.

The patient underwent both left temporal craniotomy for mesial temporal lobe tumor resection and frontal interhemispheric craniotomy for resection of the intraventricular tumor components. There were no complications intraoperatively, and the patient was transferred to the neurological intensive care unit in stable condition.

### Postoperative Course

His final pathology was consistent with RGNT. The postoperative exam was significant for central fever and autonomic dysregulation likely secondary to the tumor and its resection near the hypothalamus. Attempts were made to wean his EVD. However, a follow-up CT scan showed an interval increase in the size of the left lateral ventricle. Symptoms of hydrocephalus continued, and the patient subsequently underwent ventriculoperitoneal shunt placement.

The patient developed a proximal shunt malfunction later that month requiring revision. Gross examination demonstrated tissue buildup within the ventricular catheter fenestrations. Approximately 3 weeks later, the patient again presented with worsening hydrocephalus. Repeat revision of the proximal catheter and shunt valve was performed. At 8 months follow-up, the patient was clinically stable and back to baseline function.

## Search Strategy and Collection Criteria

A comprehensive literature review using PubMed and Web of Science was performed through November 2019 following Preferred Reporting Items for Systematic Reviews and Meta-Analyses (PRISMA) guidelines. Articles related to RGNTs were identified using the search term “rosette-forming glioneuronal tumor.” Upon removing duplicates, titles and abstracts were screened for relevant articles. English, full-text case reports and series were included. Non-English, abstract-only text, and review articles were excluded. Studies without mention of histopathological confirmation of the RGNT were further excluded. Ultimately, 80 references meeting the selection criteria were included.^1,4–82^ Patient demographics, presentations, MRI features, tumor location, treatment, and follow-up of all 130 cases (when available) were extracted and summarized. Patient presentations, genetic characteristics of tumors, recurrence analysis, and surgical follow-up are shown in Tables 1–3 and Supplementary Table 1, respectively.

**Table 1.** Presenting Symptoms

Presenting Symptom	Number of Cases	Percentage
Headache	79/112	71%
Ataxia	28/112	25%
Vomiting/nausea	27/112	24%
Visual disturbances	24/112	21%
Vertigo/dizziness	18/112	16%
Loss of consciousness	4/112	4%
Dysarthria	3/112	3%
Incidental	14/112	13%

**Table 2.** Genetics Table

	Cachia 2014^22^	Ellezam 2012^28^	Ellezam 2012^28^	Ellezam 2012^28^	Eye 2017^29^	Kitamura 2018^47^	Kitamura 2018^47^	Kitamura 2018^47^	Lin 2016^51^	Thommen 2013^74^
PIK3CA	Exon 9: G>A E545K	Exon 9: A>G E542K	Exon 20: >G H1047R	Exon 20: A>G H1047R	Exon 9: G>A E545K	Wild type	Wild type	Exon 20: A>G H1047R	Exon 20: A>G H1047R	Exon 20: A>G H1047R
FGFR1	NT	NT	NT	NT	NT	Exon 14: A>G K656E and A>G D652G	Exon 14: A>G K656E and A>G D652G	Exon 12: C>A N546K [Glial component only]	Exon 12: C>A N546K	NT

NT, not tested.

**Table 3.** Recurrence Analysis Table

Procedure	Total Cases	Number of Recurrences	Time to Recurrence	Mean Time to Recurrence	Relative % of Recurrences	Paper	Adjunctive Treatment
GTR	62	6	10 years	6.17 years	9.70%	Jacques^39^	None
			9 years			Ellezam^28^	None
			7 years			Kwon^49^	None
			4 years			Ramos^64^	None
			4 years			Ellezam^28^	None
			3 years^a^			Morris^57^	None
STR	32	3	6 years	2.92 years	9.40%	Jayapalan^40^	None
			2 years^a^			Morris^57^	Chemotherapy
			9 months			Thurston^75^	None
PR	7	1	4 months	4 months	14%	Yamamoto^6^	None
Biopsy only	4	2	6 months	3.5 months	50%	Chiba^25^	None
			1 month			Silveira^69^	None

GTR, gross total resection; PR, partial resection; STR, subtotal resection.

^a^Same patient. First received STR + chemo, then GTR after initial recurrence.

## Discussion

### Grading

The RGNT is a rare neoplasm, with 130 patients across 80 studies having been identified in the literature. RGNT is currently classified as a benign tumor,^3^ although many authors report sudden onset of aggressive behavior after prolonged periods of stability. While the most cases of RGNT have an excellent postresection prognosis without any deterioration, the outcome of our illustrative case and systematic review call this indolent nature into question. Our patient’s 6 years of MRIs demonstrate that RGNT has the potential to become rapidly progressive and infiltrative after years of dormancy. Our patient’s tumor showed significant progression on his scan an additional 3 years later. This MRI also revealed a second tumor in the foramen of Monroe. This type of progression is not unique to our case. Of the 91 cases in our review that reported follow-up, 13 demonstrated either recurrence (with or without malignant transformation) or rapid progression/dissemination, totaling 14% of the cases with reported follow-up.

However, there is much inconsistency with regard to the natural history of these tumors. Chiba et al. described a tumor that was only biopsied at first but demonstrated rapid progression of the tumor as well as a new lesion in only 6 months.^25^ A case described by Cabezas et al. presented with a secondary intraspinal lesion and leptomeningeal spread of the tumor.^21^ Silveira et al. also described the dissemination of tumor with drop metastasis in the lumbar spine only 1 month after initial imaging and biopsy of the tumor.^69^ Other reports have described similarly quick tumor recurrences postoperatively after 4 months^6^ and 9 months.^75^ However, recurrences have also been reported as late as 9 years after initial resection.^28^ Morris et al. described a particularly aggressive RGNT case in a 6-year-old boy.^57^ In this case, the tumor progressed and extended into the proximal cervical spinal cord 2 years after initial treatment involving both resection and chemotherapy.^57^ Another round of chemotherapy did not stop the tumor from further progression, so the tumor was totally resected.^57^ Unfortunately, the tumor recurred yet again 3 years later.^57^ There are even 2 cases that describe an RGNT with malignant transformation to glioblastoma after several years.^40,49^ As a greater number of cases are reported, it appears that some RGNTs may not be as indolent as previously thought.

### Epidemiology

RGNT most commonly arises in young adults, as in the case of our 19-year-old patient. However, the youngest reported patient was 4 years old, and the oldest was 81 years old.^25,26^ The mean age of diagnosis is 29.8 years, which is very similar to earlier findings.^1,11,25^ However, we noted a much less dramatic gender preference with a female:male ratio of 1.1:1 (68 females and 62 males) in contrast to previous reports describing a female predominance closer to 2:1.^1,5,42,76^

### Presenting Symptoms

Common presenting symptoms of RGNT from our review include headache (71%), ataxia/gait disturbance (25%), vomiting/nausea (24%), visual disturbances (21%), and vertigo/dizziness (16%). Several cases were also found incidentally (13%). Presenting symptoms are displayed in Table 1.

### Imaging Characteristics

The location of our patient’s tumor makes this case unique from other reports. The original lesion remained stable in the patient’s temporal lobe before progressing to involve the gangliocapsular region, bilateral thalami, tectum, and cerebellum. To our knowledge, only 2 other cases report any involvement of the temporal lobe with RGNT,^56,80^ and only 6 others had involvement of the cerebellum.^42,43,66,74,76^ The appearance of the second mass is also notable, especially with its growth into the third ventricle and foramen of Monro. While there have been 13 other reports of RGNT invasion of the third ventricle, to our knowledge, this is the first case to demonstrate invasion and blockage of the foramen of Monro.

### Genetics/Molecular Markers

The origin of RGNT has yet to be elucidated due to the rarity of the tumor and its indolent course. It has been hypothesized that the neoplasm originates from pluripotent cells of the subependymal plate,^60^ and Chakraborti et al. showed an evidence of differentiation of stem cells in their case series on RGNT.^83^ Many genetic mutations have also been found in association with RGNT. The most common genetic marker associated with RGNT is the *PIK3CA* mutation.^28,29,51,74,84,85^  *FGFR1* mutations have also been implicated in tumor pathogenesis^51,84–86^ as well as *IDH1*,^40^  *KIAA1549/BRAF* gene fusion,^20^  *PPP1R1A*,^51^ and *RNF21*.^51^

Most cases presented in the literature did not test any molecular markers, and among those that did, most did not test for the now-known genetic associations such as *PIK3CA* and *FGFR1*. However, a recent retrospective cohort study was able to obtain tissue samples of 18 different patients with RGNT for comprehensive genomic testing.^84^ Sequencing of those samples revealed that all 18 contained an *FGFR1* mutation with 13 carrying a comorbid *PIK3CA* mutation. The findings of this study confirm that constitutive activation of the FGFR signaling likely plays a role in a large portion of these tumors. Because constitutive PI_3_K pathway activation (caused by *PIK3CA* mutation) has also been implicated in several of the cases in our review as well as in the previously mentioned cohort study, RGNT may commonly be a multiple-pathway disease, which is typically more characteristic of high-grade tumors.^84^ The findings of this study were not included in our genetics summary table (Table 2) due to publication after our initial database search.

Additionally, a few cases have been found in association with neurofibromatosis type 1,^7,45,68^ as well as 2 with Noonan syndrome^44,51^ and 1 with multiple sclerosis.^70^ Interestingly, there was one case that presented 4 months after a grade II diffuse astrocytoma.^22^ Future research in this area may provide further answers about the pathogenesis of RGNT as well as direction for future drug therapy. A summary of the *PIK3CA* and *FGFR1* genetic results from the literature review is given in Table 2.

### Treatment

Currently, surgery is the mainstay of treatment. Because RGNT has historically been thought of as indolent, gross total resection (GTR) is typically only attempted if there is very low risk of damaging surrounding structures. Subtotal resection (STR), partial resection (PR), and simple biopsy with observation have all been employed for these tumors due to the risk of doing more harm with unnecessary aggressive removal.^23,37,67^ Among the cases that reported treatment (116 cases), 62 underwent GTR (53%), 32 underwent STR (28%), 7 underwent a partial resection (6%), and 4 underwent a biopsy only (3%). Other adjunctive treatments included radiotherapy (9 cases, 8%) and chemotherapy (4 cases, 3%). Though there is not a large enough sample size of recurrences to draw significant conclusions regarding the most effective treatment plan, Morris et al. postulate a pattern of delayed recurrence of tumor for patients that receive GTR as opposed to earlier recurrence for those that receive STR.^57^ Although the results of our analysis are not quite as striking, the average time to tumor recurrence for patients that underwent GTR was longer at 6.17 years compared to 2.92 years to recurrence for patients that underwent STR. However, even with this small sample size, the probability of recurrence approximates 10% with either GTR or STR. Thus, while RGNT might have a more aggressive nature than previously thought, aggressive resection in all cases may not be warranted. Based on the potential for a rapidly progressive clinical course, it is our opinion that gross total resection should be pursued when feasible. Surgical follow-up data for each case, including location and treatment, are displayed in Supplemental Table 1. Recurrence data are summarized in Table 3.

The effectiveness of chemotherapy and radiotherapy as primary or adjunctive therapy remains unclear. These therapies have very rarely been applied to RGNT treatment and typically only in cases of recurrent or disseminated tumors. We identified only one case, described by Morris et al., in which chemotherapy was not effective at limiting the rapid spread of RGNT.^57^ In this case, 4 cycles of vincristine, etoposide, and carboplatin were given after the initial resection, which resulted in stable tumor remnant for 2 years. When the tumor recurred and extended into the spinal cord, another chemotherapy regimen was attempted using weekly vinblastine for 6 months, which did not halt the progression of tumor growth.^57^ However, all other reported uses of chemotherapy (3) and all reported uses of radiotherapy (9) in cases of RGNT have yielded stable tumor remnant and no recurrence for varying lengths of follow-up.

## Conclusion

RGNT is a rare low-grade central nervous tumor usually localized within the fourth ventricle that is typically managed with surgical resection and often remains stable at follow-up. We present a case with stable tumor size and character on imaging for 3 years and sudden progression with a development of a secondary lesion 3 years later. Upon review of the literature, there are many examples of rapid progression, recurrences, and even malignant transformation, calling RGNT’s indolent reputation into question. There is insufficient evidence to support adjunctive treatment such as chemotherapy and radiotherapy in prevention of recurrence, but GTR and STR seem to have similar effectiveness as measured by patient survival and tumor recurrence. Further research is necessary to uncover the pathogenesis and genetic basis of RGNT so that better targeted therapies for aggressive cases that may be developed.

## Supplementary Material

vdaa116_suppl_Supplementary_Table_1
