# Supplementary material for: Rosette-forming glioneuronal tumor: an illustrative case and a systematic review
Source: Neurooncol Adv. 2020 Sep 9;2(1):vdaa116. doi: 10.1093/noajnl/vdaa116 (PMC7586144; doi:10.1093/noajnl/vdaa116)
Supplement: vdaa116_suppl_Supplementary_Table_1 [file vdaa116_suppl_supplementary_table_1.docx]

| **Surgical Follow-Up** | | | | | | | |
| --- | --- | --- | --- | --- | --- | --- | --- |
| **Paper** | **Sex** | **Age** | **Location** | **Treatment** | **Surgical Approach** | **F/U Status** | **F/U Length (months)** |
| Komori 2002^1^ | M | 25 | 4th ventricle, aqueduct, pineal region | None | Endoscopic biopsy | N/a | N/a |
| Komori 2002^1^ | F | 59 | 4th ventricle, aqueduct | STR + radiotherapy | N/a | Dead | 45 |
| Komori 2002^1^ | F | 24 | 4th ventricle, aqueduct, pineal region, vermis, midbrain, lt. thalamus | STR | N/a | No recurrence | 24 |
| Komori 2002^1^ | M | 18 | 4th ventricle, aqueduct | GTR | N/a | No recurrence | 24 |
| Komori 2002^1^ | F | 40 | Lower 4th ventricle, vermis | GTR | N/a | No recurrence | 6 |
| Komori 2002^1^ | F | 38 | Lower 4th ventricle | GTR | N/a | No recurrence | 16 |
| Komori 2002^1^ | F | 39 | 4th ventricle, aqueduct | GTR | N/a | No recurrence | 24 |
| Komori 2002^1^ | M | 27 | 4th ventricle | STR | N/a | No recurrence | 19 |
| Komori 2002^1^ | F | 18 | 4th ventricle, aqueduct | STR | N/a | No recurrence | 12 |
| Komori 2002^1^ | M | 46 | 4th ventricle, vermis, right cerebellum, dorsal pons | STR | N/a | No recurrence | 3 |
| Komori 2002^1^ | F | 12 | Tectum, pineal region, aqueduct | STR | N/a | No recurrence | 2 |
| Adachi 2005^12^ | F | 18 | 4th ventricle | STR | Suboccipital craniotomy through a midline scalp  incision | No recurrence | 48 |
| Albanese 2005^13^ | F | 32 | 4th ventricle | GTR | Suboccipital craniotomy | No recurrence | 9 |
| Jacques 2006^39^ | M | 39 | 4th ventricle | GTR | Unspecified craniotomy | No recurrence | 20 |
| Jacques 2006^39^ | F | 33 | Pineal gland, quadrigeminal cistern, 4th ventricle, pontine tegmentum, left  cerebellar peduncles | GTR | Posterior fossa craniectomy | Recurrence | 120 |
| Recurrence | | | N/a | GTR | Posterior fossa craniectomy | N/a |  |
| Jacques 2006^39^ | M | 42 | 4th ventricle | GTR | Posterior fossa craniectomy | N/a | N/a |
| Johnson 2006^42^ | F | 29 | 4th ventricle, inferior cerebellum | STR | N/a | N/a | N/a |
| Arai 2010^17^ | F | 15 | 4th ventricle | GTR | Transvermian approach | No recurrence | 36 |
| Vajtai 2007^76^ | F | 16 | 4th ventricle, cerebellum | GTR | Suboccipital paramedian craniotomy | N/a | N/a |
| Vajtai 2007^76^ | F | 30 | 4th ventricle | GTR | Suboccipital craniotomy | N/a | N/a |
| Marhold 2008^55^ | M | 20 | 4th ventricle, upper cerebellar midline, aqueduct | GTR | Infratentorial–supracerebellar approach | No recurrence | 21 |

| Marhold  2008^55^ | F | 47 | Lower vermis, 4th ventricle | GTR | N/a | N/a | N/a |
| --- | --- | --- | --- | --- | --- | --- | --- |
| Marhold  2008^55^ | F | 39 | Left flocculus | GTR | N/a | No recurrence | 2 |
| Marhold  2008^55^ | M | 35 | 4th ventricle | STR | N/a | No recurrence | 24 |
| Pimentel  2008^60^ | F | 38 | 4th ventricle | STR | N/a | No recurrence | 18 |
| Pimentel  2008^60^ | F | 51 | 4th ventricle | GTR | Suboccipital craniotomy | No recurrence | 8 |
| Tan 2008^72^ | M | 42 | Cerebral aqueduct | None | Endoscopic biopsy | No recurrence | 24 |
| Tan 2008^72^ | F | 38 | Vermis | None | Biopsy via suboccipital craniotomy | No recurrence | 14 |
| Anan 2009^9^ | F | 44 | Cervicothoracic spinal cord | GTR | C4 to T4 laminectomy, midline approach | No recurrence | 14 |
| Gao 2009^32^ | F | 27 | 4th Ventricle | GTR | Suboccipital craniotomy | No recurrence | 10 |
| Joseph 2009^43^ | F | 38 | 4th ventricle, cerebellum | GTR | Midline suboccipital craniectomy | No recurrence | 36 |
| Joseph 2009^43^ | M | 24 | 4th ventricle, left cerebellar tonsil, vermis. | PR | Midline suboccipital craniectomy | No recurrence | 6 |
| Li 2009^50^ | M | 27 | 4th ventricle | GTR | N/a | N/a | N/a |
| Scheithauer 2009^7^ | M | 23 | Left optic chaism | STR | Left frontotemporal craniotomy | No recurrence | 60 |
| Wang 2009^77^ | F | 16 | 4th ventricle, lateral ventricle | PR + radiotherapy + shunting 5 months later | N/a | No recurrence | 7 |
| Frydenberg  2010^30^ | M | 29 | Pineal Gland | GTR | Suboccipital transtentorial craniotomy | N/a | N/a |
| Ghosal 2010^35^ | M | 22 | Pineal gland, tectal region, thalamus | Decompression | Suboccipital transtentorial craniotomy | N/a | N/a |
| Kinno 2010^46^ | F | 18 | 4th ventricle | STR | N/a | N/a | N/a |
| Kinno 2010^46^ | M | 18 | cerebellar vermis | PR | N/a | N/a | N/a |
| Luan 2010^52^ | F | 30 | 4th ventricle | GTR | Right suboccipital retrosigmoidal approach | No recurrence | 9 |
| Rainov 2010^63^ | M | 22 | Middle, upper vermis | STR | Posterior fossa craniotomy | No recurrence | 12 |
| Shah 2010^66^ | F | 10 | vermis | GTR | N/a | No recurrence | 84 |

| Shah 2010^66^ | F | 41 | 4th ventricle, cerebellum | GTR | N/a | No recurrence | 72 |
| --- | --- | --- | --- | --- | --- | --- | --- |
| Shah 2010^66^ | F | 59 | 4th ventricle | GTR | N/a | N/a | N/a |
| Shah 2010^66^ | F | 16 | Vermis | GTR | N/a | No recurrence | 3 |
| Shah 2010^66^ | F | 17 | Vermis | GTR | N/a | No recurrence | 18 |
| Shah 2010^66^ | F | 6 | Cerebellum | GTR | N/a | N/a | N/a |
| Fushimi 2011^31^ | F | 28 | 4th ventricle | STR | Suboccipital craniotomy | No recurrence | 24 |
| Gessi 2011^34^ | M | 18 | Cerebellar Vermis | GTR | N/a | N/a | N/a |
| Karafin 2011^44^ | M | 18 | 4th ventricle | GTR | N/a | No recurrence | 21 |
| Podlesek  2011^61^ | M | 70 | 4th ventricle | GTR | Suboccipital median craniotomy | No recurrence | 24 |
| Sharma 2011^67^ | F | 16 | Tectal region | Biopsy + radiotherapy | Biopsy: CT guided stereotactic biopsy was done via a transfrontal route | N/a | 6 |
| Sharma 2011^67^ | M | 17 | Supresellar region involving hypothalamus  & 3rd ventricle | Biopsy + shunting | Frontotemporal craniotomy | N/a | N/a |
| Solis 2011^5^ | F | 16 | Pineal region | STR | Suprasellar, infratentorial approach | No recurrence | 2 |
| Alturkustani  2012^16^ | M | 16 | 4th ventricle | PR | N/a | Increased enhancement after 23 years, followed by death due to suicide | 300 |
| Ellezam 2012^28^ | F | 29 | 4th ventricle, Inferior Vermis | GTR | N/a | No recurrence | N/a |
| Ellezam 2012^28^ | F | 23 | 4th ventricle, Inferior Vermis | GTR | N/a | No recurrence | N/a |
| Ellezam 2012^28^ | M | 12 | 4th ventricle, Inferior Vermis | GTR | N/a | Recurrence 9 years later | 120 |
| Recurrence |  |  | 4th ventricle, Inferior Vermis | GTR | N/a | Stable 1 year later |  |
| Ellezam 2012^28^ | M | 50 | 4th ventricle, Inferior Vermis | N/a | N/a | N/a | N/a |
| Ellezam 2012^28^ | M | 45 | Midbrain, tectal region | GTR | N/a | No recurrence | N/a |
| Ellezam 2012^28^ | F | 18 | 4th ventricle, Inferior Vermis | GTR | N/a | Recurrence 4 years later with sacral, interventricular dissemination | 72 |
| Recurrence | |  | 4th ventricle, Inferior Vermis | Chemotherapy | N/a | Stable 2 years later |  |
| Ellezam 2012^28^ | F | 30 | 3rd ventricle | GTR | N/a | No recurrence | N/a |
| Ellezam 2012^28^ | M | 15 | 4th ventricle, Inferior Vermis | GTR | N/a | No recurrence | N/a |
| Gessi 2012^33^ | F | 27 | 4th ventricle | N/a | N/a | Recurrence | 12 |

| Gessi 2012^33^ | M | 54 | 4th ventricle | N/a | N/a | N/a | N/a |
| --- | --- | --- | --- | --- | --- | --- | --- |
| Gessi 2012^33^ | F | 42 | 4th ventricle | N/a | N/a | N/a | N/a |
| Gessi 2012^33^ | M | 9 | 4th ventricle | N/a | N/a | N/a | N/a |
| Gessi 2012^33^ | M | 32 | 4th ventricle | N/a | N/a | N/a | N/a |
| Gessi 2012^33^ | M | 12 | 4th ventricle | N/a | N/a | N/a | N/a |
| Gessi 2012^33^ | F | 34 | 3rd ventricle | N/a | N/a | N/a | N/a |
| Gessi 2012^33^ | F | 13 | 4th ventricle | N/a | N/a | N/a | N/a |
| Hsu 2012^38^ | M | 42 | 4th ventricle | PR | Occipital craniotomy | No recurrence | 18 |
| Kemp 2012^45^ | M | 33 | Lateral ventricle | GTR | Intraventricular transcallosal approach | N/a | N/a |
| Xiong 2012^4^ | M | 38 | Septum pellucidum, extending into 3rd ventricle | STR | Transcortical approach from superior and middle frontal gyrus to right ventricle | No recurrence | 6 |
| Xu 2012^79^ | M | 39 | Pineal gland, tectum, 3rd ventricle | GTR + ETV | Stereotactically guided suboccipital transtentorial  craniotomy | No recurrence | 42 |
| Yin 2012^81^ | F | 21 | 4th ventricle | N/a | N/a | N/a | N/a |
| Alnaami 2013^15^ | M | 57 | 3rd ventricle | None | Endoscopic biopsy | No recurrence | 6 |
| Alnaami 2013^15^ | M | 28 | 3rd ventricle | N/a | Endoscopic biopsy | N/a | N/a |
| Damodaran  2013^26^ | F | 81 | 4th ventricle | GTR | Posterior fossa craniotomy with transvermian approach | No recurrence | 4 |
| Kumar 2013^48^ | F | 22 | 4th ventricle | GTR | Suboccipital midline craniotomy | No recurrence | 12 |
| Thommen  2013^74^ | M | 74 | Cerebellum | N/a | N/a | N/a | N/a |
| Thurston  2013^75^ | F | 8 | 4th ventricle | STR | N/a | Recurrence 9 months later | 10 |
| Recurrence | |  | 4th ventricle | STR | N/a | Stable 6 weeks later |  |
| Xiong 2013^78^ | M | 23 | Left anterior cingulate cortex | GTR | Left frontal craniotomy | No recurrence | 8 |
| Zhang 2013^82^ | M | 20 | 4th ventricle | STR + radiotherapy | Suboccipital craniotomy with partial inferior transvermian  approach | No recurrence | 89 |
| Zhang 2013^82^ | M | 49 | 4th ventricle | STR | Suboccipital craniotomy with telovelar approach | No recurrence | 43 |

| Cachia 2014^22^ | F | 36 | Tectum, vermis | STR | N/a | N/a | N/a |
| --- | --- | --- | --- | --- | --- | --- | --- |
| Chiba 2014^25^ | F | 4 | Tectal area, pineal region, 4th ventricle | GTR | Occipital transtentorial approach | No recurrence | 8 |
| Chiba 2014^25^ | F | 19 | Tectal area | Biopsy | N/a | Recurrence | 6 |
| Recurrence |  |  | Tectal area | GTR | Midline occipital craniotomy | N/a |  |
| Matyja 2014^56^ | M | 22 | Temporal lobe | GTR | Left pterional craniotomy | No recurrence | 44 |
| Allinson 2015^14^ | F | 33 | 4th ventricle | N/a | Endoscopic biopsy | N/a | N/a |
| Bidinotto  2015^20^ | M | 33 | Cervicothoracic spinal cord | GTR | C6-T4 laminectomy | No recurrence | 52 |
| Cabezas 2015^21^ | M | 24 | First lesion: 4th ventricle, cervical spinal cord (metastisis) | Biopsy + chemotherapy + radiotherapy | Biopsy: craniectomy of the posterior fossa | Malignant course, but no recurrence | 24 |
| Haryu 2015^37^ | F | 24 | Tectum | Biopsy + ETV | N/a | No recurrence | 17 |
| Maiti 2015^53^ | M | 12 | 3rd Ventricle | GTR | Midline suboccipital craniectomy, supracerebellar infratentorial approach | No recurrence | 9 |
| Nair 2015^58^ | M | 15 | 4th ventricle | STR | Midline suboccipital craniectomy + C1 arch excision | N/a | N/a |
| Ogut 2015^59^ | F | 29 | 4th ventricle | N/a | N/a | N/a | N/a |
| Simmons  2015^70^ | F | 27 | 4th ventricle, cerebral aqueduct | Biopsy, ETV | N/a | N/a | N/a |
| Yamamoto  2015^6^ | F | 8 | Hypothalamus | PR | Anterior transcallosal approach with septostomy | Recurrence 4 mo later | 36 |
| Recurrence | | | Hypothalamus | PR + radiotherapy | N/a | Stable 3 years later |  |
| Beuriat 2016^19^ | F | 13 | Cerebellar Hemisphere | STR | N/a | N/a | N/a |
| Cebula 2016^23^ | F | 75 | Thalamus, 3rd ventricle | None | Endoscopic biopsy | No recurrence | 12 |
| Chen 2016^24^ | F | 21 | 3rd ventricle, 4th ventricle, suprasellar region | STR | N/a | Dead | N/a |
| Eastin 2016^27^ | F | 33 | 3rd Ventricle, Thalamus | N/a | N/a | N/a | N/a |
| Hakan 2016^36^ | M | 29 | 4th ventricle | STR + ETV | N/a | No recurrence | 32 |
| Lin 2016^51^ | F | 12 | 4th ventricle | STR | Suboccipital craniotomy | No recurrence | 16 |
| Makita 2016^54^ | F | 55 | 3rd Ventricle | GTR | Interhemispheric trans lamina terminalis approach | No recurrence | 27 |
| Sieg 2016^68^ | F | 41 | Tectum | Shunting + STR 5 years later | Suboccipital transvermian craniotomy | Dead | 7 |

| Bera 2017^18^ | M | 16 | 4th ventricle | GTR | Midline posterior fossa craniectomy, vermian split  approach | No recurrence | 12 |
| --- | --- | --- | --- | --- | --- | --- | --- |
| Duan 2017^11^ | F | 26 | Thoracolumbar spinal cord | GTR | Midline approach with T9-T11 laminectomy. | No recurrence | 15 |
| Duan 2017^11^ | F | 35 | Cervicothoracic spinal cord | GTR | C4-C6 laminectomy | No recurrence | 17 |
| Eye 2017^29^ | M | 35 | 3rd Ventricle, Thalamus | STR | N/a | No recurrence | 12 |
| Morris 2017^57^ | M | 6 | 4th ventricle | STR + chemotherapy | Suboccipital craniotomy | Progression and extension into proximal cervical spinal cord 2 years later | 84 |
| 1st recurrence | | | 4th ventricle, cervical spinal cord | Chemotherapy | N/a | Further progression and extension 6 months later |  |
| 2nd recurrence | | | 4th ventricle, cervical spinal cord | GTR | N/a | Recurrence 3 years later |  |
| 3rd recurrence | | | 4th ventricle | None | Endoscopic biopsy | Stable 2 years later |  |
| Pradhan 2017^62^ | M | 21 | Cerebellar vermis | GTR | Midline suboccipital craniotomy | No recurrence | 24 |
| Collin 2018^10^ | F | 40 | Cervical spinal cord | GTR + shunting | Median laminectomy | No recurrence | 6 |
| Kitamura  2018^47^ | F | 15 | 4th ventricle, vermis | STR | N/a | No recurrence | 24 |
| Kitamura  2018^47^ | M | 55 | Left lateral ventricle | GTR | N/a | No recurrence | 26 |
| Kitamura  2018^47^ | M | 9 | Left occipital lobe | GTR | N/a | No recurrence | 60 |
| Kitamura  2018^47^ | M | 30 | Tegmentum mesencephali | PR | N/a | No recurrence | 36 |
| Kitamura  2018^47^ | F | 67 | Tectum mesencephali | None | Endoscopic biopsy | No recurrence | 37 |
| Kitamura  2018^47^ | M | 19 | Right frontal lobe | GTR | N/a | No recurrence | 3 |

| Ramos 2018^64^ | M | 32 | 4th ventricle | GTR | Midline suboccipital craniectomy | Recurrence 4 years later | 84 |
| --- | --- | --- | --- | --- | --- | --- | --- |
| Recurrence | | | 4th ventricle | Radiotherapy | N/a | Stable 3 years later |  |
| Sumitomo  2018^71^ | M | 9 | Right parietal lobe | GTR, resection of the cingulate gyrus and superior parietal lobe | N/a | N/a | N/a |
| Yapicier 2018^80^ | F | 55 | Hippocampus, mesial temporal lobe | GTR | N/a | N/a | N/a |
| Bharadwaj 2019^8^ | M | 12 | suprasellar cistern | Radiotherapy | Endoscopic biopsy | No recurrence | N/a |
| Jayapalan  2019^40^ | M | 42 | 4th ventricle | STR | N/a | Recurrence with transformation to glioblastoma | 72 |
| Jiménez-  Heffernan  2019^41^ | M | 42 | 4th ventricle | N/a | N/a | N/a | N/a |
| Kwon 2019^49^ | M | 58 | Left cerebellar hemisphere | GTR | N/a | Recurrence 7 years later with transformation to glioblastoma | 84 |
| Recurrence | | | Left cerebellar hemisphere | GTR + chemotherapy | N/a | N/a |  |
| Sekar 2019^65^ | M | 18 | Opticochiasmatic region | STR | Craniotomy | No recurrence | 6 |
| Silveira 2019^69^ | M | 49 | 4th ventricle + drop metastasis in the lumbar spine + dissemination to the other ventricles, hypothalamus and pituitary  infundibulum | None | Endoscopic biopsy | Metastatic course with recurrence 1 month later | 9 |
| Recurrence | | | 4th ventricle | STR + shunting + radiotherapy | Posterior fossa craniectomy with C1 posterior arch removal | N/a |  |
| Tanaka 2019^73^ | F | 18 | Pons | None | Biopsy via midline suboccipital craniotomy | No recurrence | 204 |
| Present Study | M | 19 | Left temporal lobe, left  gangliocapsular region, bilateral thalami,  tectum, cerebellum | GTR | Left temporal craniotomy and frontal interhemispheric craniotomy | No recurrence | 8 |

F/U = Follow-up; N/a = Information not available; GTR = Gross total resection; STR = Subtotal resection; PR = Partial resection
